# Comparison of pregnancy and neonatal outcomes in a retrospective full pregnancy history survey versus population-based prospective records: a validation study in rural Sarlahi District, Nepal

**DOI:** 10.1186/s41043-023-00472-5

**Published:** 2023-12-08

**Authors:** Daniel J. Erchick, Tsering P. Lama, Seema Subedi, Andrea Verhulst, Michel Guillot, Subarna K. Khatry, Steven C. LeClerq, James M. Tielsch, Luke C. Mullany, Joanne Katz

**Affiliations:** 1 Department of International Health, Johns Hopkins Bloomberg School of Public Health, 615 N. Wolfe Street, Baltimore, MD 21205 USA; 2 Nepal Nutrition Intervention Project – Sarlahi, Kathmandu, Nepal; 3 Centre d’Estudis Demogràfics (CED-CERCA), Barcelona, Spain; 4 University of Pennsylvania, Philadelphia, PA USA; 5 Department of Global Health, Milken Institute School of Public Health, George Washington University, Washington, DC USA; 6 Applied Physics Laboratory, Johns Hopkins University, Baltimore, MD USA; 7 French Institute for Demographic Studies (INED), Aubervilliers, France

## Abstract

**Introduction:**

Countries without complete civil registration and vital statistics systems rely on retrospective full pregnancy history surveys (FPH) to estimate incidence of pregnancy and mortality outcomes, including stillbirth and neonatal death. Yet surveys are subject to biases that impact demographic estimates, and few studies have quantified these effects. We compare data from an FPH vs. prospective records from a population-based cohort to estimate validity for maternal recall of live births, stillbirths, and neonatal deaths in a rural population in Sarlahi District, Nepal.

**Methods:**

We used prospective data, collected through frequent visits of women from early pregnancy through the neonatal period, from a population-based randomized trial spanning 2010–2017. We randomly selected 76 trial participants from three pregnancy outcome groups: live birth (*n* = 26), stillbirth (*n* = 25), or neonatal death (*n* = 25). Data collectors administered the Nepal 2016 Demographic and Health Surveys (DHS)-VII pregnancy history survey between October 22, 2021, and November 18, 2021. We compared total pregnancy outcomes and numbers of pregnancy and neonatal outcomes between the two data sources. We matched pregnancy outcomes dates in the two sources within ± 30 days and calculated measures of validity for adverse outcomes.

**Results:**

Among 76 participants, we recorded 122 pregnancy outcomes in the prospective data and 104 outcomes in the FPH within ± 30 days of each woman’s total observation period in the trial. Among 226 outcomes, we observed 65 live births that survived to 28 days, 25 stillbirths, and 32 live births followed by neonatal death in the prospective data and participants reported 63 live births that survived to 28 days, 15 stillbirths, and 26 live births followed by neonatal death in the pregnancy history survey. Sixty-two FPH outcomes were matched by date within ± 30 days to an outcome in prospective data. Stillbirth, neonatal death, higher parity, and delivery at a health facility were associated with likelihood of a non-matched pregnancy outcome.

**Conclusions:**

Stillbirth and neonatal deaths were underestimated overall by the FPH, potentially underestimating the burden of mortality in this population. There is a need to develop tools to reduce or adjust for biases and errors in retrospective surveys to improve reporting of pregnancy and mortality outcomes.

**Supplementary Information:**

The online version contains supplementary material available at 10.1186/s41043-023-00472-5.

## Introduction

In 2020, more than 5 million children under the age of five died, with almost half occurring in the first 28 days of life (neonatal deaths) [1]. The United Nations established Sustainable Development Goal 3 to address the burden of preventable deaths among children, including an aim of reducing neonatal deaths to < 12 per 1,000 live births by 2030 [2–4]. Collecting accurate data on live births, stillbirths, and neonatal and child deaths is critical for generation of mortality estimates to track progress toward global, regional, and national goals.

Complete civil registration and vital statistics systems (CRVS systems) that collect prospective data on vital events are the ideal source of such mortality data [5]. Yet countries without strong CRVS systems often generate mortality estimates from retrospective full birth histories or full pregnancy histories collected through sample surveys, such as the United States Agency for International Development (USAID)-supported Demographic and Health Surveys (DHS) and Unicef-supported Multiple Indicator Cluster Surveys (MICS) [6, 7]. However, pregnancy and birth history surveys are subject to biases that can impact demographic estimates, including stillbirth and neonatal mortality rates [8, 9].

The DHS-VIII woman’s questionnaire reproductive section includes an full pregnancy history with questions about pregnancy characteristics, outcomes, and timing, including for live births, miscarriages, abortions, stillbirths, and neonatal deaths, spanning the entire pregnancy history [10]. The long period of recall, difficulties in distinguishing between stillbirths and neonatal deaths, and sensitive nature of these questions present risks for various biases. These include omissions of pregnancy outcomes [9], which may lead to underestimates of fertility or mortality; date displacement [11], which can shift a pregnancy outcome or death outside the reference period; age errors [11], which can shift a death into a different mortality period; and misclassification of stillbirths and neonatal deaths [12], which can occur in either direction for a variety of reasons. Omissions of stillbirths and neonatal deaths are an important limitation of DHS surveys, often with higher likelihood of occurring early in the neonatal period [9]. These issues demonstrate the heavy reliance of the DHS full pregnancy histories estimates of stillbirth and neonatal mortality on accurate maternal recall. Hence, the measurement of the validity of pregnancy history survey instruments is critical but difficult to obtain given the absence of high-quality “gold standard” or reference data with which to make comparisons.

The goal of this study is to compare pregnancy and neonatal mortality outcome data from a full pregnancy history survey against prospectively collected data from a population-based randomized trial that utilized high-frequency follow-up of women in pregnancy through the infant’s first 28 days of life. Specifically, we will compare numbers of pregnancy and neonatal outcomes and calculate measures of validity for live birth that survived to 28 days, stillbirth, and neonatal death events.

## Methods

In this study, we compare data on pregnancy and neonatal outcomes collected through a full pregnancy history survey questionnaire against prospectively collected data in a sub-area of Sarlahi District, Nepal. This study was conducted at a population-based field site for maternal, newborn, and child health and nutrition research operated by the Nepal Nutrition Intervention Project Sarlahi (NNIPS) since 1989. Specifically, we evaluate the following research aims:

1. Compare numbers of pregnancy and neonatal outcomes and other characteristics reported in the full pregnancy history survey to those recorded in the prospective data.
2. Compare, among pregnancy outcomes matched by delivery date (± 30 days) in the two data sources, measures of agreement and validity for pregnancy and neonatal outcomes, using the prospective data as the reference.

### Prospective data

We used prospectively collected data from a large, population-based randomized trial of topical applications for newborn massage, the Nepal Oil Massage Study (NOMS) (NCT01177111), which enrolled and followed pregnant women and their infants in 34 Village Development Committees (VDCs) in Sarlahi District, Nepal, between November 2010 and January 2017. At the start of the trial, data collectors conducted a census activity to systematically visit all households in the study area to update existing project maps and database. During the trial, data collectors monitored all married women 15–35 years through a pregnancy surveillance system. Local-resident-female data collectors visited women every 5 weeks to ask about the date of last menstrual period (LMP) and offer a pregnant test if needed. Pregnant women were enrolled in the study and followed monthly in pregnancy, as soon as possible after delivery, and through the neonatal period with visits on days 1, 3, 7, 10, 14, 21, and 28. The trial recorded 32,114 live births, 865 stillbirths, and 998 neonatal deaths.

The trial collected baseline data on participants’ demographic characteristics, socioeconomic status, and pregnancy history. At the study visit following delivery, data collectors recorded the date/time of the pregnancy outcome, location of delivery, labor and delivery characteristics, health status of the mother and infant, and infant anthropometry (weight, length, head circumference, temperature). Data collectors asked participants if each pregnancy outcome was a miscarriage, abortion, live birth only, stillbirth only, or live birth(s) and stillbirth(s) (in cases of multiple gestation). We made a final classification between live birth and stillbirth based upon three questions: Did the baby ever cry? Did the baby ever move? Did the baby ever breathe? If the mother or caregiver answered yes to any of these questions, we classified the outcome as live birth; otherwise, we classified it as a fetal loss. We made a classification between miscarriages (< 28 weeks) and stillbirths (≥ 28 weeks) among fetal losses using the gestational age at delivery calculated from the LMP date, which was obtained at the time of enrollment, early in pregnancy. Data collectors administered a stillbirth verbal autopsy module to the mother or caregiver for this outcome. In cases of death of a liveborn infant, data collectors administered a neonatal verbal autopsy module.

### Pregnancy history survey data

We randomly selected participants from an eligible list of women from the population-based trial participants, including 25 individuals from three pregnancy and neonatal outcome groups: live birth that survived to 28 days (*n* = 26), stillbirth (*n* = 25), or live birth followed by neonatal death (*n* = 25). We enrolled one extra live birth than intended due to a logistical error and opted to retain the participant’s data for analysis. We defined eligibility as a woman who had a singleton pregnancy outcome between January 1, 2015, and July 20, 2017, in 23 VDCs (Additional file 1: Table S1). This provided us a recall period of an average of about 5 years. About 80% of women in the trial were visited within 72 h of delivery, and we also restricted to this group to minimize the time between the occurrence of the pregnancy outcome and the trial data collection. If a potential participant could not be contacted, another woman with the same outcome was randomly selected from the list of eligible participants. Data collectors then visited selected participants to request participation, conduct consent, and administer the Nepal 2016 Demographic and Health Surveys (DHS)-VII pregnancy history questions (Additional file 1: Table S2). The pregnancy history questionnaire included questions to document all previous pregnancies beginning with the first (forward history), including miscarriages, stillbirths, live births; single or multiple birth, duration of pregnancy (in weeks or months); infant sex, infant name, infant vital signs (cry, move, or breathe), infant death, and age at death (in days, weeks, or months).

### Comparison of pregnancy and neonatal outcomes

We compared participant characteristics, as recorded in the prospective data, stratified by the three pregnancy and neonatal outcome groups using Chi-squared tests among the 76 women enrolled in the pregnancy history survey. To compare pregnancy and neonatal outcomes in the two data sources, we included all pregnancy outcomes in the prospective data observed among the 76 enrolled women (which occurred between May 25, 2011, and May 25, 2017) and pregnancy outcomes reported in the pregnancy history survey that occurred during the prospective follow-up period for each participant plus 30 days before and after their observation. Specifically, this period was defined for each individual participant as the time from 30 days before their enrollment date in the prospective study and 30 days after either the last birth visit or last pregnancy surveillance visit (whichever was later) in the prospective study. This yielded an analytic dataset, referred to as the “complete dataset,” of 226 pregnancy outcomes, including 122 from the prospective data and 104 from the pregnancy history survey.

### Validity analysis

We aimed to match the known pregnancy outcome dates in the prospective data (*n* = 122) to the pregnancy outcome dates reported in the pregnancy history survey (*n* = 104). We addressed partially recalled dates in the pregnancy history survey by assigning a value of 15 for missing day and June for missing month. We calculated the difference in days between the events in the prospective and pregnancy history survey for each participant using the 226 outcomes in the “complete dataset.” We generated an analytic dataset, referred to as the “matched dataset,” of 62 matched pregnancy outcomes (*n* = 124 total), that occurred within ± 30 days. We defined a non-matched pregnancy outcome as an outcome in the prospective data with no pregnancy outcome reported in the pregnancy history survey within ± 30 days of the known pregnancy outcome date. The match was only determined using pregnancy outcome dates because of the high level of missing name data and high discrepancy in sex between the two sources. In this community, it is common to delay naming of an infant until several weeks after birth. Our data collectors are trained to enter “baby boy” or “baby girl” when no name had yet been provided by the family. This occurred often in the trial as most participants were visited within 72 h of birth. We also conducted a sensitivity analysis to match dates with different ranges, including ± 60 days, ± 100 days, ± 365 days, and for all outcomes without restriction, and then compared pregnancy outcomes in the two data sources for each match. For matches >  ± 100 days, some women reported multiple outcomes in the pregnancy history survey that matched with a single outcome from the prospective data; in these cases, we considered the matched outcomes as those with the closest set of dates.

We presented a table to compare agreement by date, sex, and pregnancy and neonatal outcomes stratified by pregnancy and neonatal outcome according to the prospective data. We assessed the individual validity of the three pregnancy and neonatal outcomes reported in the pregnancy history survey by estimating sensitivity, specificity, positive predictive value, negative predictive value, and proportion correctly classified for each outcome. We used a logistic regression model with generalized estimating equations, to account for correlation associated with reporting of ≥ 1 singleton pregnancies for each woman, to estimate adjusted odds ratios and 95% confidence intervals of non-matched pregnancy outcomes by participant characteristics.

We graphed pregnancy outcomes that did not match within ± 30 days from both data sources (*n* = 317) by participant. In an attempt to determine the reason for failure to match by date and outcome type (live birth, stillbirth, or neonatal death), we classified each pregnancy outcome in the prospective data (*n* = 60) as one of the following potential error categories by comparing it to the outcomes from the pregnancy history survey for each participant: 1) date error or omission; 2) misclassification or omission; 3) date error, misclassification, or omission; 4) definite omission. More details on this analysis can be found in Additional file 1: Fig. S1.

### Stillbirth and neonatal outcome counts

We plotted live births that survived to 28 days, stillbirths, and live births followed by neonatal death by time of birth for each participant according to the two data sources among pregnancy outcomes in the complete dataset. We presented the following from each data source: total pregnancy outcomes, median pregnancy outcomes per participant, duration and time between pregnancies, and numbers of pregnancy and neonatal outcomes. Specifically, we compared the number of stillbirths and neonatal deaths reported in the complete dataset of 226 pregnancy outcomes, including the 122 outcomes from the prospective data and 104 outcomes from the pregnancy history survey, and the date matched dataset of 124 pregnancy outcomes, including 62 outcomes from each source.

### Ethical approval

The trial and the pregnancy history survey received ethical approval from the Johns Hopkins Bloomberg School of Public Health Institutional Review Board, Baltimore, MD, USA. The trial received ethical approval from the Institutional Review Board of Tribhuvan University Institute of Medicine, Kathmandu, Nepal, and the pregnancy history survey received ethical approval from the Ethical Review Board of the Nepal Health Research Council (Kathmandu, Nepal).

## Results

### Participant selection

**Fig. 1:**
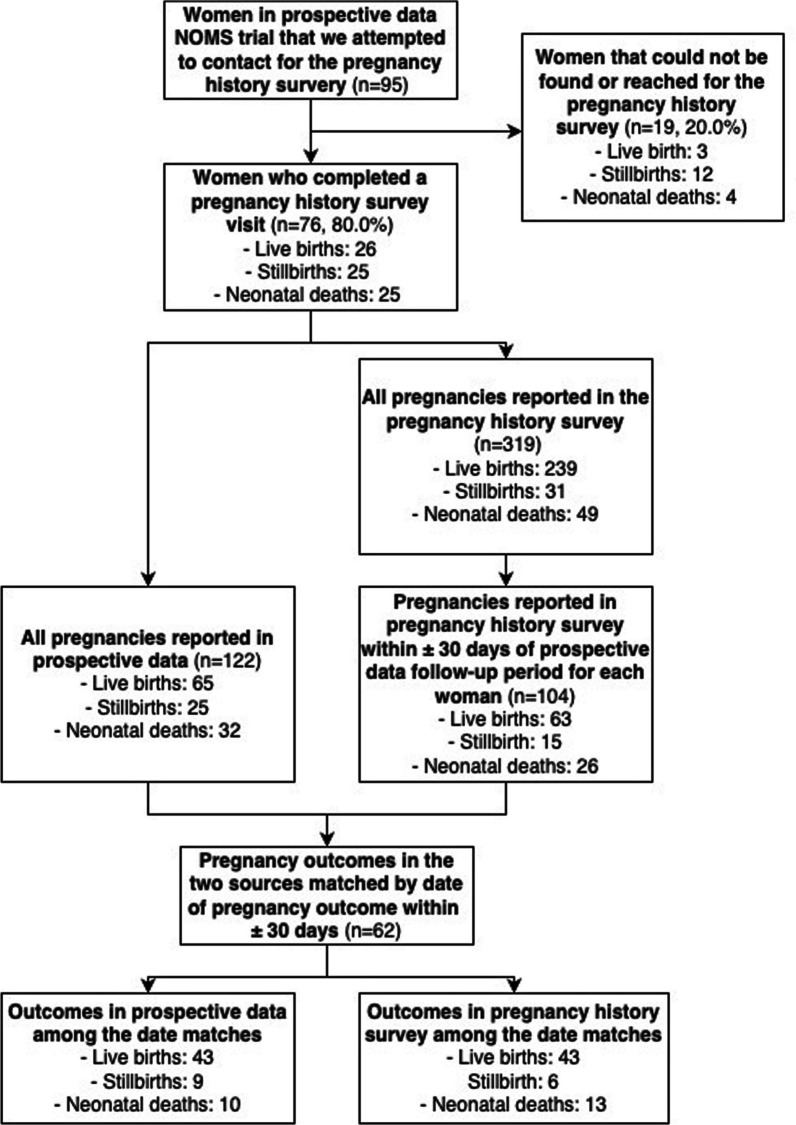
Participant flowchart and outcomes by data source

We contacted 95 potential participants from the trial, of whom 19 (20.0%) could not be contacted or had moved away from the study area, and 76 (80.0%) were met in-person, consented, and enrolled into the pregnancy history survey between October 22, 2021, and November 18, 2021 (Fig. 1). Across all pregnancy outcomes, there were no significant differences in participant characteristics between the 76 enrolled participants and the 19 participants that could not be contacted (Additional file 1: Table S3). The proportion that could not be contacted varied by outcome: live birth (*n* = 3/29, 10.3%), stillbirth (*n* = 12/37, 32.4%), and neonatal death (*n* = 4/29, 13.8%) groups (Chi-squared test, *p* = 0.072). The 76 participants enrolled in the pregnancy history survey included women with at least one live birth that survived to 28 days (*n* = 26), stillbirth (*n* = 25), or live birth followed by neonatal death (*n* = 25), according to the prospective data, between January 4, 2015, and May 25, 2017. All participants in the trial were visited within 72 h after each of these pregnancy outcomes (median: 1 day).

The characteristics of the 76 enrolled participants are presented in Table 1. There were no significant differences in participant characteristics by the three pregnancy outcome categories. However, women who experienced a stillbirth had lower literacy than those who experienced a live birth that survived to 28 days or live birth followed by neonatal death combined (illiteracy rate: live birth/neonatal death: *n* = 19/51, 37.3%, stillbirth: *n* = 3/25, 12.0%; *p* = 0.023).

**Table 1:** Participant characteristics by infant outcome from NOMS trial prospective data

Characteristic* ~	Live birth that survived to 28 days	Stillbirth	Live birth followed by neonatal death	*p* value
*Age*				
< 19	10 (38.5)	6 (24.0)	12 (48.0)	
20–24	10 (38.5)	11 (44.0)	6 (24.0)	
25–29	4 (15.4)	6 (24.0)	4 (16.0)	
30–34	2 (7.7)	2 (8.0)	3 (12.0)	0.618
*Ethnicity*				
Madeshi	26 (100.0)	25 (100.0)	24 (96.0)	
Pahadi	0 (0.0)	0 (0.0)	1 (4.0)	0.356
*Parity*				
0	10 (38.5)	4 (16.0)	10 (40.0)	
1	7 (26.9)	4 (16.0)	3 (12.0)	
2	2 (7.7)	9 (36.0)	5 (20.0)	
3	2 (7.7)	4 (16.0)	2 (8.0)	
4+	5 (19.2)	4 (16.0)	5 (20.0)	0.226
*Education*				
No education	17 (65.4)	22 (88.0)	17 (68.0)	
Some education	9 (34.6)	3 (12.0)	8 (32.0)	0.137
*Literacy*				
No	17 (65.4)	22 (88.0)	15 (60.0)	
Yes	9 (34.6)	3 (12.0)	10 (40.0)	0.068
*Place of delivery*				
Not in a health facility	10 (38.5)	11 (50.0)	11 (44.0)	
Facility delivery	16 (61.5)	11 (50.0)	14 (56.0)	0.724

*All data presented in this table are from the NOMS trial

~Variable missingness: Delivery location: *n* = 3 (3.9%)

### Number of pregnancy outcomes

In the prospective data, the 76 participants had a total of 122 pregnancy outcomes (median: 2, range: 1 to 4) between May 25, 2011, and May 25, 2017, during their follow-up in the NOMS trial (Table 2). In the pregnancy history survey, 76 participants reported having a total of 319 lifetime pregnancy outcomes (median: 4, range 1–11). Of the 319 pregnancy outcomes, 104 (median: 2, range 1–5) occurred within a time period for each participant spanning 30 days prior to the enrollment date and 30 days later than the last pregnancy surveillance or birth visit in the prospective data. Among the 76 participants, five had no pregnancy outcome dates in the pregnancy history survey within this time period. Of the 76 women, 56 (73.7%) reported the same number of pregnancy outcomes in both data sources, while 13 (17.1%) reported one fewer, 6 (7.9%) one more, and 1 (1.3%) two more than the pregnancy history in the time period defined. The time between the date of the 122 pregnancy outcomes in the prospective data and the date of the pregnancy history survey administration ranged from 4.4 years to 10.5 years (median 6.2).

**Table 2:** Pregnancy outcomes recorded in prospective data and pregnancy history survey

	Prospective data	Pregnancy history survey
Indicator		
Total pregnancy outcomes	122	104
Median (range) outcomes/participant	2 (1–4)	2 (1–5)
Live births that survived to 28 days (*n*, %)*	65 (53.3%)	63 (60.6%)
Stillbirths (*n*, %)*	25 (20.5%)	15 (14.4%)
Live birth followed by neonatal death (*n*, %)*	32 (26.2%)	26 (25.0%)
Median (IQR) pregnancy duration (months)	8.9 (6.4–10.3)	9 (5–9)
Median (IQR) time between pregnancies (years)	1.5 (0.9–2.6)	1.4 (0.9–2.6)

*Chi-squared test for comparison of the overall distribution of the three outcomes between prospective data and pregnancy history survey was *p* = 0.421

### Completeness of pregnancy outcome dates

All pregnancy outcome dates in the prospective data were fully recorded. In the pregnancy history survey, one-third of women (*n* = 29/76, 38.2%) were able to recall full dates for each of their pregnancy outcomes, including a specific day, month, and year, using the Nepali calendar. The other two-thirds (*n* = 47/76, 61.8%) recalled a partial date, either only the month and year or only the year, for at least one of their pregnancy outcomes. Among women who recalled at least one partial date, the number of pregnancy outcomes with a partial date ranged from 1 to 10. Of the 76 women, over half (*n* = 45/76, 59.2%) had at least one partial date due to non-recall of the day of birth (i.e., they were able to recall only the month and year), while ten percent (*n* = 8/76, 10.5%) had at least one partial date due to non-recall of the day and month (i.e., they were able to recall only the year). There were no observations with missing month but non-missing day and year; neither were there observations with missing year but non-missing day and month. In total, one-third (*n* = 108, 33.9%) of the 319 outcomes reported in the pregnancy history survey had a partial date. Of the 108 partial dates, 96 (88.9%) were missing only day and 12 (11.1%) both day and month. Among the 104 pregnancy outcomes from the pregnancy history survey that fell within the prospective observation period, and hence were available for matching, 41 (39.4%) of outcomes had a partial date, including most (*n* = 36, 87.8%) that were missing only day and 5 (12.2%) both day and month. Outcomes reported in the pregnancy history survey had different proportions of partial dates. Among the 319 outcomes, stillbirths (*n* = 25/31, 80.7%) and live births followed by neonatal death (*n* = 40/49, 81.6%) were more likely to have a partial date compared to live births that survived to 28 days (43/239, 18.0%) (Chi-squared test, *p* =  < 0.001). Among the 108 partial dates, stillbirths (*n* = 6/25, 24.0%) were more likely to have both missing day and month (rather than only missing day) compared to live births that survived (*n* = 2/43, 4.7%) and live births followed by neonatal death (*n* = *n* = 4/40, 10.0%) (Chi-squared test, *p* = 0.048).

### Pregnancy outcome date match

**Fig. 2:**
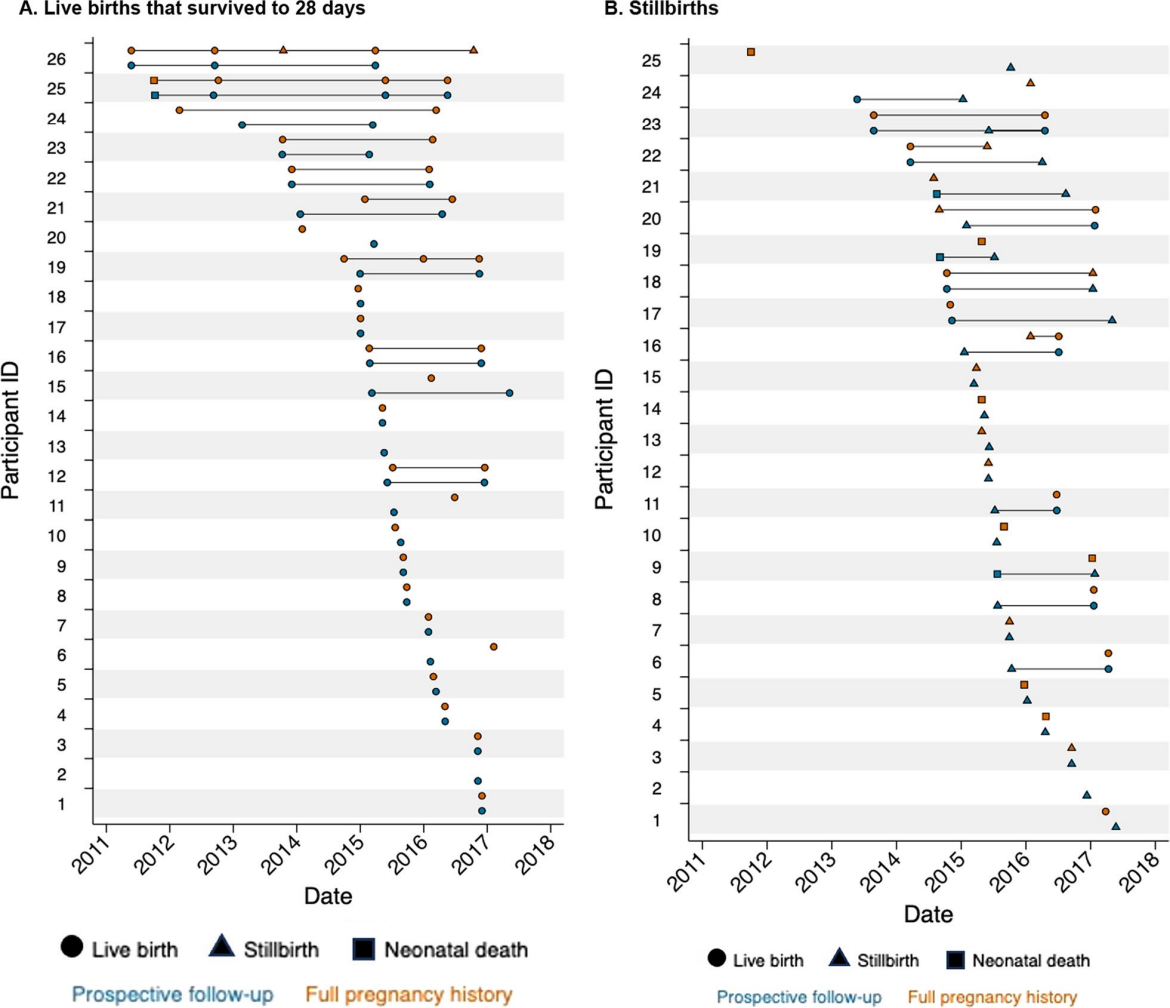

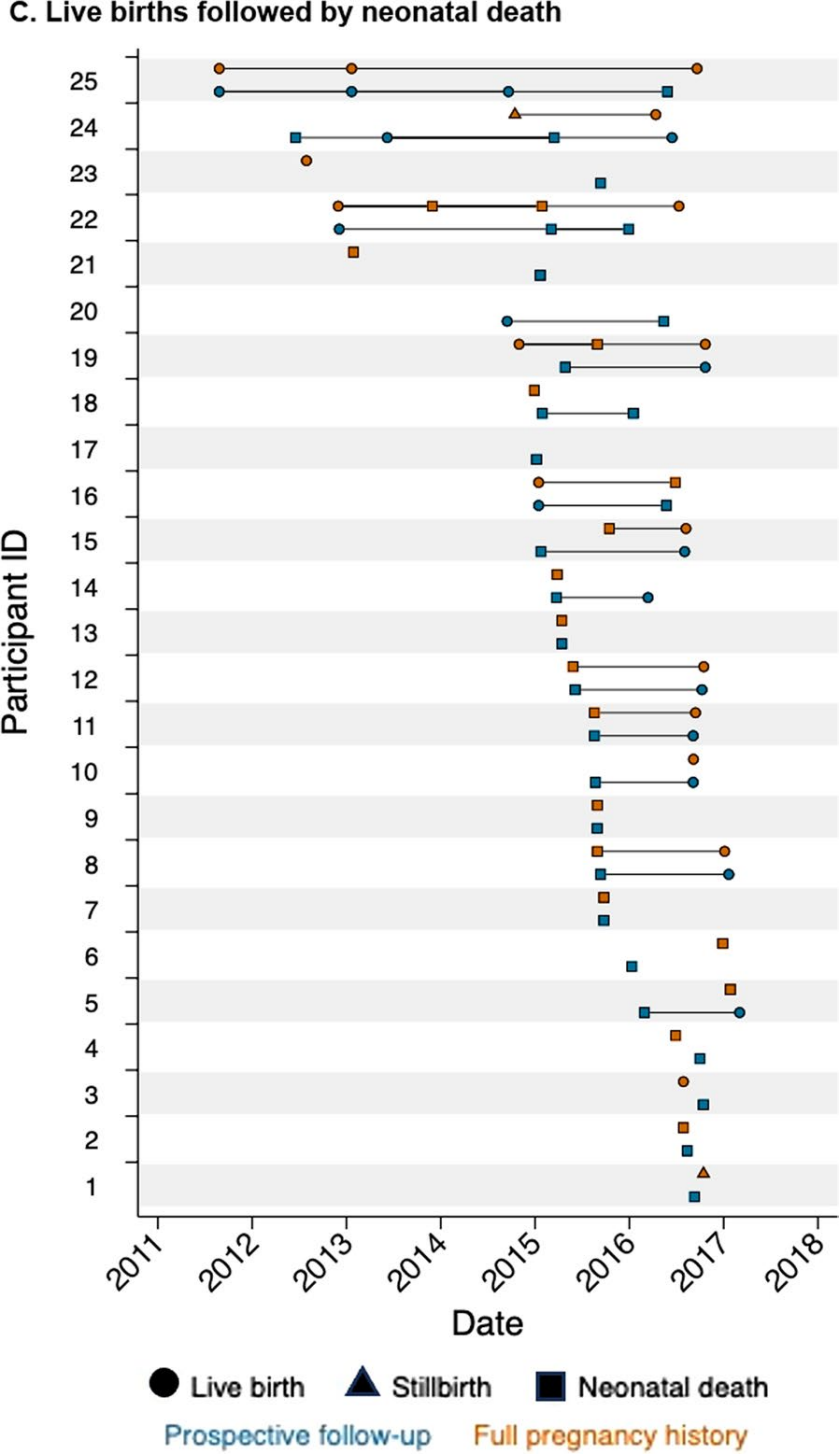
**Pregnancy outcomes from prospective data and pregnancy history survey**. The﻿ graphs below display pregnancy outcomes from the prospective data and pregnancy history survey that occurred within the prospective follow-up period for each participant plus 30 days before and after their observation. Specifically, this period was defined as the time from 30 days before the participant’s enrollment date in the prospective study and 30 days after either the last birth visit or last pregnancy surveillance visit (whichever was later) in the prospective study. This yielded 226 pregnancy outcomes, including 122 from the prospective data and 104 from the pregnancy history survey. We display pregnancy outcomes that did not match by date with ± 30 days, including those inside and outside the prospective follow-up range defined above, in Additional file 1: Fig. S1. The 76 participants enrolled in the pregnancy history survey included women with at least one live birth that survived to 28 days (*n* = 26), stillbirth (*n* = 25), or live birth followed by neonatal death (*n* = 25), according to the prospective data, between January 4, 2015, and May 25, 2017

We graphed all pregnancy outcomes from the complete dataset (*n* = 226) in Fig. 2, separated for clarity by the three pregnancy outcomes groups selected for inclusion in the validity study. Comparison of dates from the 122 and 104 pregnancy outcomes in the complete dataset resulted in 202 comparisons for date matching (Table 3). Of these, 30 (14.9%) dates matched exactly in the prospective and pregnancy history survey, while another 32 dates matched within ± 30 days, together constituting the 62 matched pregnancy outcomes in the matched dataset (*n* = 124 total). The differences between matched pregnancy outcome dates (prospective data minus pregnancy history survey) ranged from -28 to 17 days (Additional file 1: Fig. S2). The proportion of date matches by outcome was higher for live births that survived to 28 days (*n* = 43/65, 66.2%) than stillbirths (*n* = 9/25, 36.0%) or live births followed by neonatal death (*n* = 10/32, 31.3%) (*p* = 0.001). Stillbirth, neonatal death, higher parity, and delivery at a health facility were associated with occurrence of a non-matched pregnancy outcome (Table 4). The likelihood of matching did not vary by time from the pregnancy outcome in the prospective data to the date of the pregnancy history survey administration (non-match: mean 6.2 years vs. match: mean 6.4 years, t-test *p*-value: 0.649). Pregnancy outcomes that did not match within ± 30 days from both data sources are displayed in Additional file 1: Fig. S1. Results from a sensitivity analysis comparing pregnancy outcomes in both sources among outcomes matched by date with wider ranges (± 60 days, ± 100 days, etc.) is given in Additional file 1: Table S5.

**Table 3:** Measures of agreement and validity for infant outcomes for prospective data and pregnancy history survey

Pregnancy history survey data	Prospective data			*p* value
	Live birth that survived to 28 days	Stillbirth	Live birth followed by neonatal death	
*Difference in pregnancy outcome dates between data sources (n = 202)*				
≤ 30 days (*n* = 62)	43 (25.0)	9 (31.0)	10 (20.0)	
> 30- < 100 days (*n* = 16)	6 (4.9)	4 (13.8)	6 (0.1)	
≥ 100 days (*n* = 124)	74 (60.2)	16 (55.2)	34 (0.7)	
Sex match (*n* = 62)*^+^				
Match	43 (100.0)	3 (75.0)	9 (100.0)	
Not match	0 (0.0)	1 (25.0)	0 (0.0)	0.071
Validity outcome data (n = 62)*				
Live birth, survived > 28 days	43 (100.0)	0 (0.0)	0 (0.0)	
Stillbirth	0 (0.0)	5 (55.6)	1 (10.0)	
Neonatal death	0 (0.0)	4 (44.4)	9 (90.0)	**0.001**
Validity measures (n = 62)*~				
Sensitivity (%)	100.0	55.6	90.0	
Specificity (%)	100.0	98.1	92.3	
Positive predictive value (%)	100.0	83.3	69.2	
Negative predictive value (%)	100.0	92.9	98.0	
Proportion correctly classified (%)	100.0	91.9	91.9	

*p* < 0.05 are shown in bold

*For date matches ± 1 month

^+^Six newborns had no sex reported in the pregnancy history survey (3 boys, 3 girls)

~For each outcome group versus the other two groups

**Table 4:** Adjusted odds ratios and 95% confidence intervals of non-matched pregnancy outcome* for participant characteristics using logistic regression with generalized estimating equations~

Outcome (*n* = 112)	Odds ratio and 95% CI
Live birth that survived to 28 days	Ref
Stillbirth	**4.17 (1.38, 12.64)**
Live birth followed by neonatal death	**5.97 (2.16, 16.51)**
Age (years)	0.95 (0.86, 1.06)
*Parity*	
0	Ref
≥ 1	**3.45 (1.14, 10.47)**
*Education*	
No education	Ref
Some education	0.79 (0.29, 2.16)
*Antenatal care*	
< 4 visits	Ref
≥ 4 visits	0.89 (0.37, 2.21)
*Delivery location*	
Home	Ref
Health facility	**2.77 (1.16, 6.62)**
*Infant sex*	
Male	Ref
Female	1.06 (0.46, 2.41)

Confidence interval for odds ratio does not cross 1.00

*Non-matched pregnancy outcome is defined as no pregnancy outcome reported in the pregnancy history survey within ±30 days of each outcome in the prospective data

~Multivariable logistic regression model with generalized estimating equations, to account for correlation associated with reporting of ≥1 singleton pregnancies for each woman, to estimate adjusted odds ratios and 95% confidence intervals of non-matched pregnancy outcomes by participant characteristics

### Infant sex and name match

Among 62 pregnancy outcomes with a matched date, most (*n* = 55/62, 88.7%) had the same sex in both sources, although 3 (4.8%) boys and 3 (4.8%) girls had no sex recalled in the pregnancy history survey. Among the 76 selected pregnancy outcomes, one-fifth (*n* = 15/76, 19.7%) had a name match between the pregnancy history survey and the prospective data. In the pregnancy survey history data, among the non-matched names (*n* = 61/76, 80.3%), over half (*n* = 37/61, 60.7%) were listed as either “baby” or blank, while the remainder (*n* = 24/61, 39.3%) had a name. Similarly, in the prospective data, the non-matched names were mostly (*n* = 52/61, 85.2%) “baby boy _” or “baby girl _” with many fewer having a full name (*n* = 9/61, 14.8%).

### Stillbirth and neonatal outcome counts and measures of validity

Among the 226 outcomes in the complete dataset, a similar number of live births that survived to 28 days (*n* = 63/65, 96.9%) were reported in both sources, but fewer stillbirths (*n* = 15/25, 60%) and live births followed by neonatal death (*n* = 26/32, 81.3%) were reported in the pregnancy history survey compared to the prospective data (Table 2).

Among the date matched dataset of 124 outcomes, only half (*n* = 5/9, 55.6%) of stillbirths were correctly classified; the misclassified were reported as live births followed by neonatal death. The neonatal deaths were mostly correctly classified (*n* = 9/10, 90.0%); the one misclassified was reported as a stillbirth. Associations between misclassification and participant characteristics are given in Additional file 1: Table S4.

Age at death for the 10 neonatal deaths among the 62 matches ranged from 0.02 to 27.1 days (median 2.2 days) according to the prospective data. The difference in dates of death for the 9 correctly classified neonatal deaths ranged from − 2.0 days to 1.0 day (pregnancy history survey minus prospective data).

Among the 60 prospective non-date matched outcomes, the most likely errors, according to the criteria outlined in Additional file 1: Fig. S1, were date error or omission (*n* = 45, 75.0%), misclassification, or omission (*n* = 10, 16.7%), and date error, misclassification, or omission (*n* = 5, 8.3%). There were no prospective non-date matched outcomes that could be definitively classified as omissions.

## Discussion

Few studies have compared pregnancy dates and outcomes from the DHS full pregnancy history survey to prospective, population-based data at aggregate or individual levels. Our validity study, conducted in a rural Nepali community, found that a DHS full pregnancy history survey reported a similar number of live births that survived to 28 days but fewer stillbirths and live births followed by neonatal death, compared to prospectively collected trial data. Other studies have similarly reported that births of children who died were less likely to be reported in survey data than in longitudinal estimates generated from regular home visits at Health and Demographic Surveillance Systems (HDSS) sites, although these sites differ from population-based cohort studies like our trial in important ways [13, 14]. Of note, a study in Uganda that compared HDSS to a pregnancy history survey identified more pregnancies in the pregnancy history survey for recall of outcomes in the year prior and fewer pregnancies for longer recall periods [15].

The Nepal DHS 2016 neonatal mortality rate estimate of 30 deaths per 1,000 live births for Nepal’s Province 2 is similar to that observed in the prospective trial data over the 2010–2017 follow-up period (31.2). However, the NMR in the study population decreased during this period from 34.7 in 2011 to 27.5 in 2016 [16]. Our results compare with three studies that used longitudinal estimates through regular home visits at HDSS sites in Bangladesh, Uganda, and Guinea-Bissau that found higher neonatal mortality than in birth (Guinea-Bissau) or pregnancy (Bangladesh and Uganda) history surveys for various reasons [13, 15, 17]. A comparison of HDSS and pregnancy history survey data at five sites found that mortality rates were not substantially different for most sites and provided evidence that both data sources may underestimate mortality estimates for reasons that varied by data source and site [14].

About half of the 122 prospectively observed pregnancy outcomes in our study were matched by date within one month in the pregnancy history survey. The high proportion of unmatched dates could be due in part to the large number of partial dates reported in the pregnancy history survey. This, in turn, could be a result of the long recall times (median 6.2 years, range: 4.4–10.5 years) between the prospective outcome and the pregnancy history survey administration date, which is longer than the average recall in the five years preceding the survey range used by DHS. Due to high missingness of infant names or other identifying data for matching participants between these two data sources, there was no definitive method for differentiating omissions from date displacements. However, visual observation of the pregnancy outcomes in both sources that did not match within ± 30 days (Additional file 1: Fig. S1) suggests most were likely date errors or omissions (three-quarters), followed by misclassifications or omissions (17%), with the remainder difficult to classify as either date error, misclassification, or omission (8%).

Among pregnancy outcomes matched by date, live births that survived to 28 days were well classified; however, four of nine stillbirths were misreported as live births followed by neonatal death and one in ten neonatal deaths as stillbirths. This misclassification of stillbirths as neonatal deaths resulted in a neonatal mortality rate higher than the prospective data within the outcomes matched by date. Sensitivity analyses allowing for matching of pregnancy outcome dates within wider date ranges (i.e., ± 60 days, ± 100 days, ± 365 days, and unrestricted), found generally lower, but not markedly different agreement, for pregnancy outcomes in the two data sources as the date ranges widened. Again, it must be noted that a high proportion of mismatched outcomes could be due to the large number of partial dates reported in the pregnancy history survey. Underreporting and misclassification of stillbirths and neonatal deaths have been observed in other settings. A study in Bangladesh comparing HDSS data and a DHS pregnancy history survey from the 1990s found that the completeness of neonatal death reporting was 83% [13]. A study in Malawi from the 2010s showed that a full birth history survey misclassified 20% of stillbirths as neonatal deaths, but no misclassification in the other direction, compared to a detailed verbal/social autopsy survey [12]. A study in Guinea-Bissau from the 2010s compared HDSS data against a full birth history survey and found misclassification of stillbirths and neonatal deaths in both directions [17].

Examination of participant characteristics provided some insights into the drivers of reporting errors in this population. Women with higher parity in our study were more likely to omit or displace an outcome compared to the prospective data, which was similar to the study in Bangladesh that found higher parity was associated with missed live birth in surveys [13]. The study in Malawi reported an association with older age and misclassification of stillbirths and neonatal deaths, and there was some indication that this might have occurred in our data, although the numbers were too small to say definitively [12]. The study of five HDSS sites showed that socio-cultural factors, including cultural and religious beliefs, stigma, and women-specific barriers such as vulnerability of younger women, influence underreporting of adverse pregnancy outcomes [18]. A future study with a larger sample and addition of qualitative data collection would be useful in further describing the demographic, socioeconomic, and cultural factors associated with misreporting in this population.

Women who delivered at a health facility in our study were more likely to not report an outcome in the pregnancy history survey that matched with the prospective data. A study in this same population in Sarlahi District observed lower recall reliability for receipt of several labor and delivery and immediate newborn care interventions among women who delivered at a health facility compared to those that delivered at home [19]. The authors hypothesized that either the receipt of these interventions was less salient events for women delivering in facilities, e.g., due to poor counseling, unfamiliar environments, or because they paid less attention due to trust in skilled attendants, or interventions were more salient events at home because women may have better rapport with home birth attendants and family, who more effectively communicated what services were delivered. Given increasing facility delivery rates in South Asia, this finding is worthy of further study.

Age at death was fairly accurate for neonatal deaths with pregnancy outcome dates that were correctly recalled, although due to our small sample, there were few late neonatal deaths that would be more subject to age errors resulting in misclassification as postnatal deaths. Date errors and age under- or over-statements are common causes of age error that can impact neonatal mortality rates. The study in Guinea-Bissau reported a large number of postnatal deaths were transferred to the neonatal period, which could lead to overestimation of the neonatal mortality rate [17]. Other studies have demonstrated the ability of heaping errors to lead to underestimation of neonatal or infant mortality [11, 14, 20].

Our study had limitations—including a small sample size, high number of partial dates in the pregnancy history survey, and a small number of pregnancy outcome date matches, yielding fewer participants for analysis and limiting the precision of our estimates—so our findings should be interpreted with caution. The long recall period, relative to the DHS five years preceding the survey time frame, may have contributed to poor date recall and subsequent failure to match more pregnancy outcome dates. An attempt to match pregnancy outcomes by name and sex in this community was not useful, given how few infants are named at birth, which is common in South Asia and other settings. There were few participants with less common but important demographic and socioeconomic characteristics, for example, higher maternal age, Pahadi ethnicity, or more education, limiting investigations of maternal recall by these factors. Both maternal recall and pregnancy outcome incidence may also differ across by factors that we did not consider in this analysis, such as seasonality of birth [21–23]. In sampling a small number of women from the prospective study with a known live birth that survived to 28 days, stillbirth, or live birth followed by neonatal death, we could not reliably calculate probabilities of these outcomes from a population sample to compare to the pregnancy history survey. We used different interviewers for the prospective and pregnancy history survey data collection, and due to the small sample, we were not able to assess for the presence of interviewer effects between the two data sources or interviewer proficiency in delivering the pregnancy history survey relative to those who administer this survey for the DHS. Although we utilized the prospective data as the reference in this analysis, we recognize the absence of a true gold standard; it is possible, for example, that trial data collectors missed some pregnancy outcomes related to our exclusion of participants not visited within 72 h of delivery, who may have been more likely to experience a stillbirth or neonatal death.

Our findings have implications for fertility and mortality estimation in the Terai region of Nepal and similar settings. Stillbirth and neonatal deaths were underestimated by the full pregnancy history survey, which would potentially misrepresent the burden of mortality in this population. This indicates a need to design and evaluate survey measurement tools and techniques to reduce biases and errors or statistical approaches to adjust for these issues.

### Supplementary Information

**Additional file 1. Supplementary Table 1**: VDC names. **Supplementary Table 2**: Pregnancy History Survey Questionnaire. **Supplementary Figure 1**: All pregnancy outcomes excluding those that matched (dates within ±30 days) from the prospective data and pregnancy history survey data sources (n=317). **Supplementary Table 3**: Participant characteristics by enrollment status in pregnancy history survey. **Supplementary Figure 2**: Difference in days between dates of pregnancy outcome for the prospective data vs. pregnancy history survey for outcomes matched within ±30 days (n=124 outcomes; n=62 per source). **Supplementary Table 4**: Misclassification of stillbirths and neonatal deaths by participant characteristics among 62 pregnancy outcomes matched by date within one month. **Supplementary Table 5**: Pregnancy outcomes in the prospective and pregnancy history survey data after matching outcomes by date within ±30 days, ±60 days, ±100 days, ±365 days, and without restriction*.

## Data Availability

Not applicable.

## Declarations

### Patients or public involvement

Patients or the public were not involved in the design, conduct, reporting, or dissemination plans of our research.
