# Supplementary material for: Comparison of pregnancy and neonatal outcomes in a retrospective full pregnancy history survey versus population-based prospective records: a validation study in rural Sarlahi District, Nepal
Source: J Health Popul Nutr. 2023 Dec 8;42:139. doi: 10.1186/s41043-023-00472-5 (PMC10709973; doi:10.1186/s41043-023-00472-5)
Supplement: Supplementary file 1 — Additional file 1. Supplementary Table 1: VDC names. Supplementary Table 2: Pregnancy History Survey Questionnaire. Supplementary Figure 1: All pregnancy outcomes excluding those that matched (dates within ±30 days) from the prospective data and pregnancy history survey data sources (n=317). Supplementary Table 3: Participant characteristics by enrollment status in pregnancy history survey. Supplementary Figure 2: Difference in days between dates of pregnancy outcome for the prospective data vs. pregnancy history survey for outcomes matched within ±30 days (n=124 outcomes; n=62 per source). Supplementary Table 4: Misclassification of stillbirths and neonatal deaths by participant characteristics among 62 pregnancy outcomes matched by date within one month. Supplementary Table 5: Pregnancy outcomes in the prospective and pregnancy history survey data after matching outcomes by date within ±30 days, ±60 days, ±100 days, ±365 days, and without restriction*. [file 41043_2023_472_MOESM1_ESM.docx]

**SUPPLEMENTARY FILE**

**Supplementary Table 1: VDC names**

| AURAHI |
| --- |
| BABARGANJ |
| BARHATHAWA |
| BELA |
| DHANGADA |
| GAMHARIYA |
| HARIPUR |
| HEMPUR |
| ISHWARPUR |
| JAMUNIYA |
| JANAKINAGAR |
| KABILASI |
| KISANPUR |
| LAUKAT |
| LAXMIPUR KODARKOT |
| MOHANPUR |
| MURTIYA |
| PHARHADAWA |
| PIDARI |
| PIPARIYA |
| SAHODAWA |
| SALEMPUR |

**Supplementary Table 2: Pregnancy History Survey Questionnaire**

**Supplementary Figure 1: All pregnancy outcomes excluding those that matched (dates within ±30 days) from the prospective data and pregnancy history survey data sources (n=317)**

In total, our study had 122 pregnancy outcomes from the prospective data and 319 pregnancy outcomes from the pregnancy history survey, for a total of 441 pregnancy outcomes. A total of 62 outcomes (124 total) in each dataset were matched by date within ±30 dates, leaving 317 outcomes that did not match by date. As the prospective data had 122 outcomes, of which 62 matched by date within ±30 days, 60 of the outcomes displayed below are from the prospective data (blue) and the remaining 257 (317 total) are from the pregnancy history survey (orange).

The graphs below display these 317 pregnancy outcomes from the pregnancy history survey that did not match within ±30 days with an outcome in the prospective data, including dates outside the range of the NOMS trial follow-up period for each participant. The 74 participants that contributed these 317 outcomes are split across three graphs by the outcome groups from Figure 2 for clarity (Panels A, B, & C). Some outcomes in the two data sources look like they are close in time and would be date matches according to our methodology, but, in fact, they are all at least 31 days apart and therefore not matched. Also included in the graphs are the prospective follow-up periods for each participant shaded in gray. Specifically, this period was defined using the prospective trial data for each individual participant from the date of the first pregnancy surveillance visit to the date of the last birth visit or last pregnancy surveillance visit (whichever was later).

In an attempt to identify/distinguish date errors, omissions, and misclassifications, we defined the following categories below. Then, individually for each of the 74 participants, we reviewed their outcomes that did not match by type and date within ±30 days, to assess which of these categories most likely applied to them. In cases where there was more than one outcome, we visually assessed all of the outcomes for the participant to determine for each prospective outcome what was the most likely error to have occurred for each prospective outcome across the categories above. We then assigned a most likely cause of error starting from the earliest prospective outcome and continuing to the latest (written left to right in the margin). There were 2 participants for which all outcomes matched; they are marked below as “No outcomes that didn’t match.”

1. **No issue:** A participant for whom all prospective pregnancy outcomes matched by date within ±30 days of a pregnancy outcome from the pregnancy history survey. These participants have no blue prospective pregnancy outcomes in the *Supplementary Figure 1, Panel A, B, and C* graphs.
2. **Date error or omission:** A participant for whom at least one pregnancy outcome in the prospective data did not match within ±30 days of an outcome from the pregnancy history survey, and there was at least one pregnancy outcome that matched by type in both data sources outside the ±30 day window. Therefore, either one of the pregnancy history survey outcomes is a true match that had an incorrectly recalled date or the outcome was omitted (in which case the outcomes reported in the pregnancy history survey are for other events).
3. **Misclassification or omissions:** A participant for whom at least one pregnancy outcome in the prospective data did not match within ±30 days of an outcome from the pregnancy history survey, and although there were no outcomes that matched by type in the pregnancy history survey, there was at least one outcome of another type. Therefore, either the one of the pregnancy history survey outcomes is a true match that was misclassified or the outcome was omitted (in which case the outcomes and dates reported in the pregnancy history survey are for other events).
4. **Date error, misclassification, or omission:** A participant for whom at least one pregnancy outcome in the prospective data did not match within ±30 days of an outcome from the pregnancy history survey, and there is evidence of a pregnancy history survey outcome matching in type *and* another outcome not matching in type that is closer in time. This suggests there could be either a date error or misclassification. Omission in this case is less likely to have occurred but is still possible.
5. **Definite omission:** A participant for whom at least one pregnancy outcome in the prospective data did not match within ±30 days of an outcome from the pregnancy history survey, but there were no other outcomes in the pregnancy history survey. This situation did not occur for any participants in our study.

**Summary of likely errors**

|  | **By participant** | | **By outcome** | |
| --- | --- | --- | --- | --- |
| **Likely error or no issue** | **No.** | **%*** | **No.** | **%*** |
| No issue | 27 | NA | 27 | NA |
| Date error or omission | 34 | 72.3% | 45 | 75.0% |
| Misclassification or omission | 6 | 12.8% | 10 | 16.7% |
| Date error, misclass., or omission | 2 | 4.3% | 5 | 8.3% |
| Definite omission | 0 | 0.0% | 0 | 0.0% |
| Multiple issues | 5 | 10.6% | NA | NA |
|  |  |  |  |  |
| **Total** | 74 |  | 87 |  |
| **Total minus "no issue"** | 47 | 63.5% | 60 | 69.0% |
| *** Denominator excludes “no issue”** | | | | |

**A. Live births that survived to 28 days**

| 26. No issue |
| --- |
| 25. No issue |
| 24. Date error or omission (2) |
| 23. Date error or omission |
| 22. No issue |
| 21. Date error or omission (2) |
| 20. Date error or omission |
| 19. Date error or omission |
| 18. No issue |
| 17. No issue |
| 16. No issue |
| 15. Date error or omission (2) |
| 15. No issue |
| 13. Date error or omission |
| 12.Date error or omission |
| 11. Date error or omission |
| 10. Date error or omission |
| 9. No issue |
| 8. No issue |
| 7 No issue |
| 6 Date error or omission |
| 5. No issue |
| 4. No issue |
| 3. No outcomes that didn’t match |
| 2. Date error or omission |
| 1. No issue |


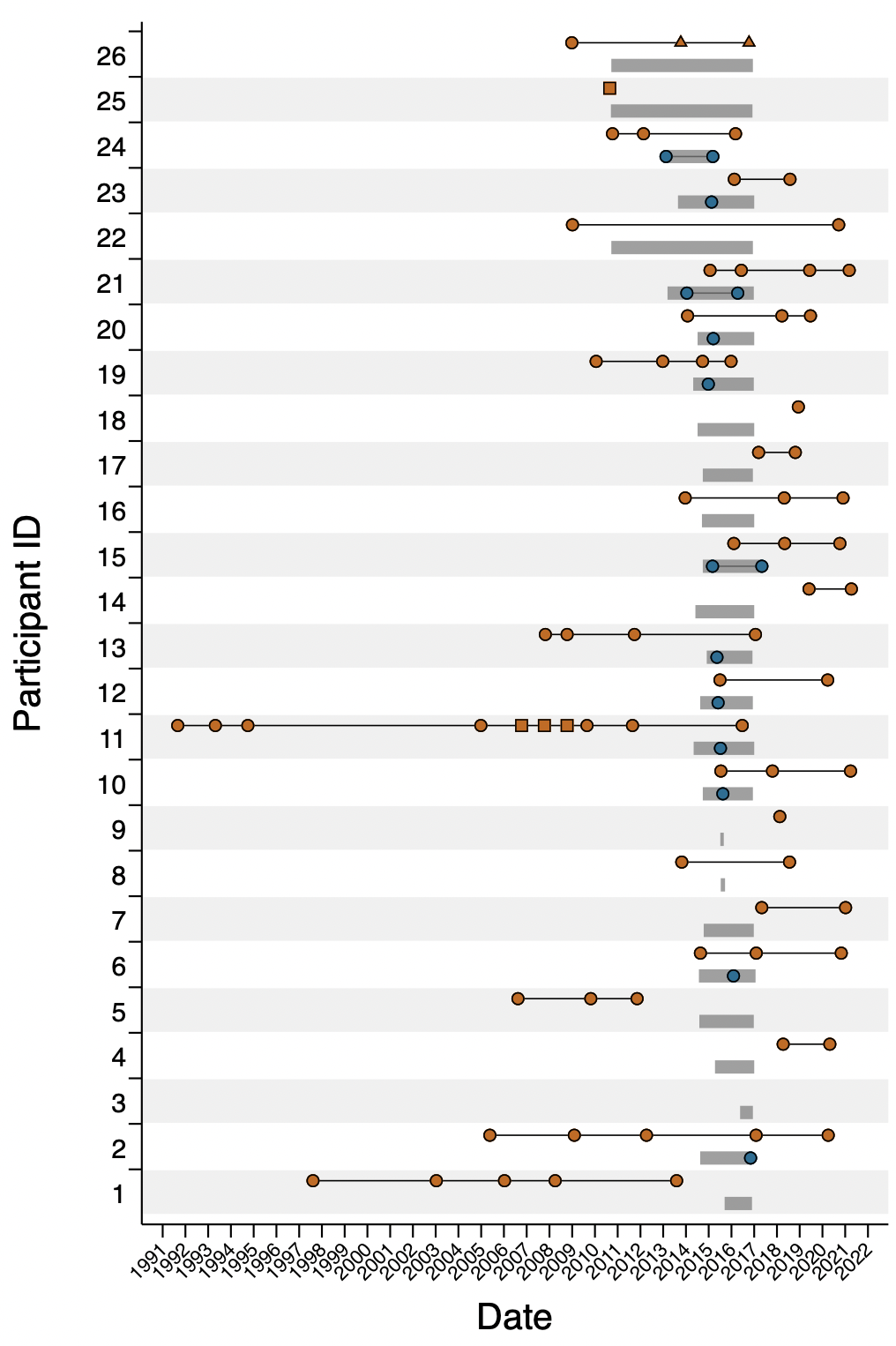


**
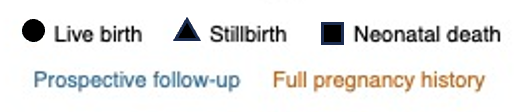

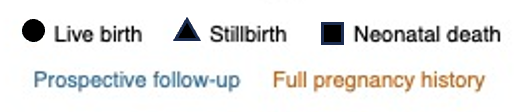
**

**B. Stillbirths**


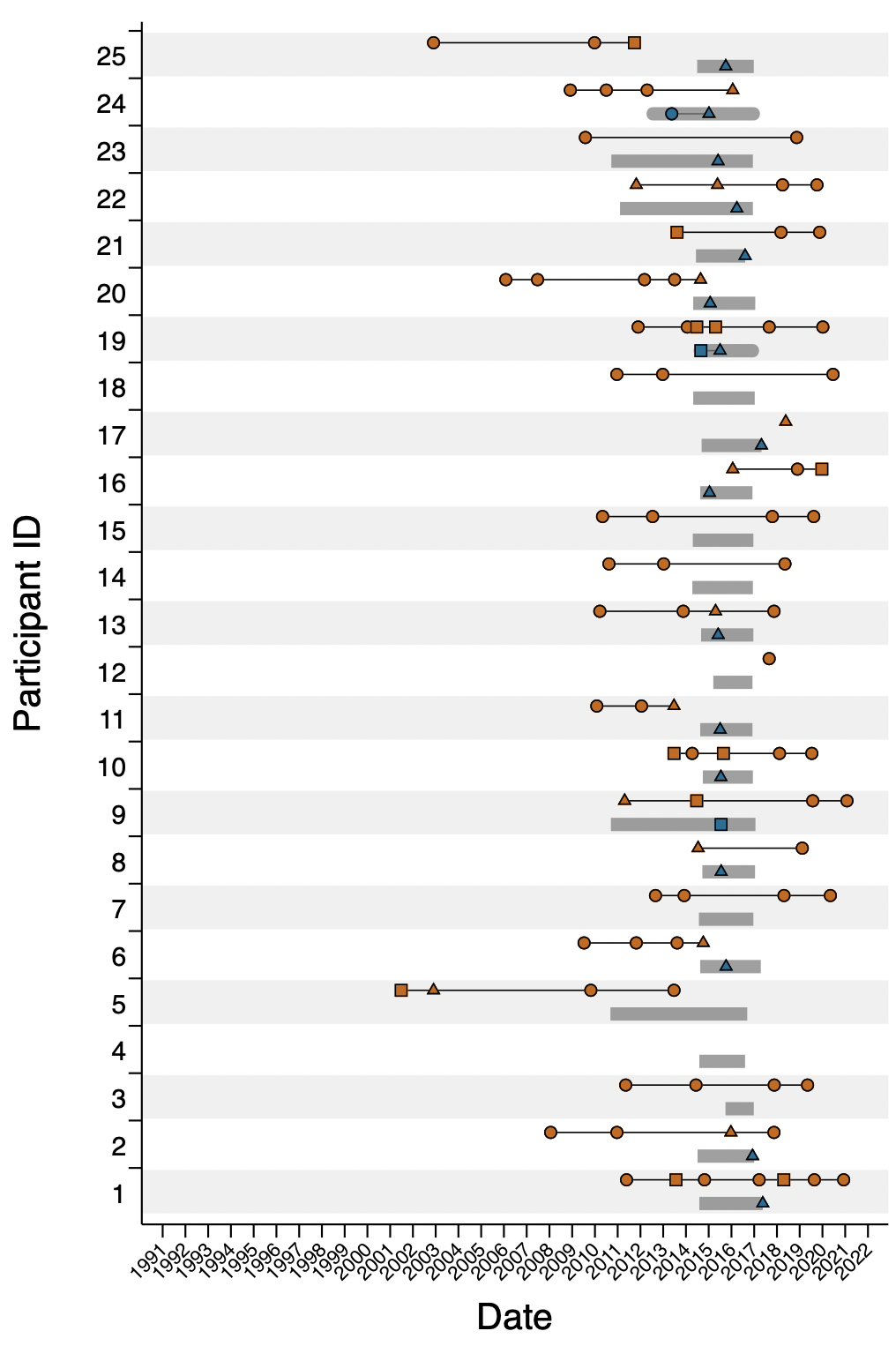


| 25. Misclassification or omission |
| --- |
| 24. Date error or omission (2) |
| 23. Misclassification or omission |
| 22. Date error or omission |
| 21. Misclassification or omission |
| 20. Date error or omission |
| 19. Date error or omission; Misclassification or omission |
| 18. No issue |
| 17. Date error or omission |
| 16. Date error or omission |
| 15. No issue |
| 14. No issue |
| 13. Date error or omission |
| 12. No issue |
| 11. Date error or omission |
| 10. Misclassification or omission |
| 9. Date error or omission |
| 8. Date error or omission |
| 7. No issue |
| 6. Date error or omission |
| 5. No issue |
| 4. No outcomes that didn’t match |
| 3. No issue |
| 2. Date error or omission |
| 1. Misclassification or omission |

**
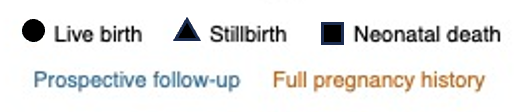

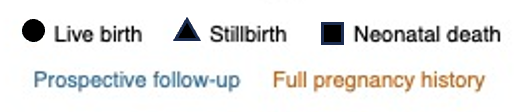
**

**C. Live births followed by neonatal death**

| 25. Date error or omission (2) |
| --- |
| 24. Misclass. or omission; date error, misclass., or omission; misclass. or omission; date error or omission |
| 23. Misclassification or omission |
| 22. Date error or omission; date error, misclass., or omission |
| 21. Date error or omission |
| 20. Date error or omission & misclass. or omission |
| 19. Date error or omission |
| 18. Date error or omission (2) |
| 17. Date error or omission |
| 16. Date error or omission |
| 15. Date error or omission |
| 14. Date error or omission |
| 13. No issue |
| 12. No issue |
| 11. No issue |
| 10. Date error or omission |
| 9. No issue |
| 8. No issue |
| 7. No issue |
| 6. Date error or omission |
| 5. Date error or omission; date error, misclass. or omission |
| 4. Date error or omission |
| 3. Date error, misclass., or omission |
| 2. No issue |
| 1. Date error, misclass., or omission |

**
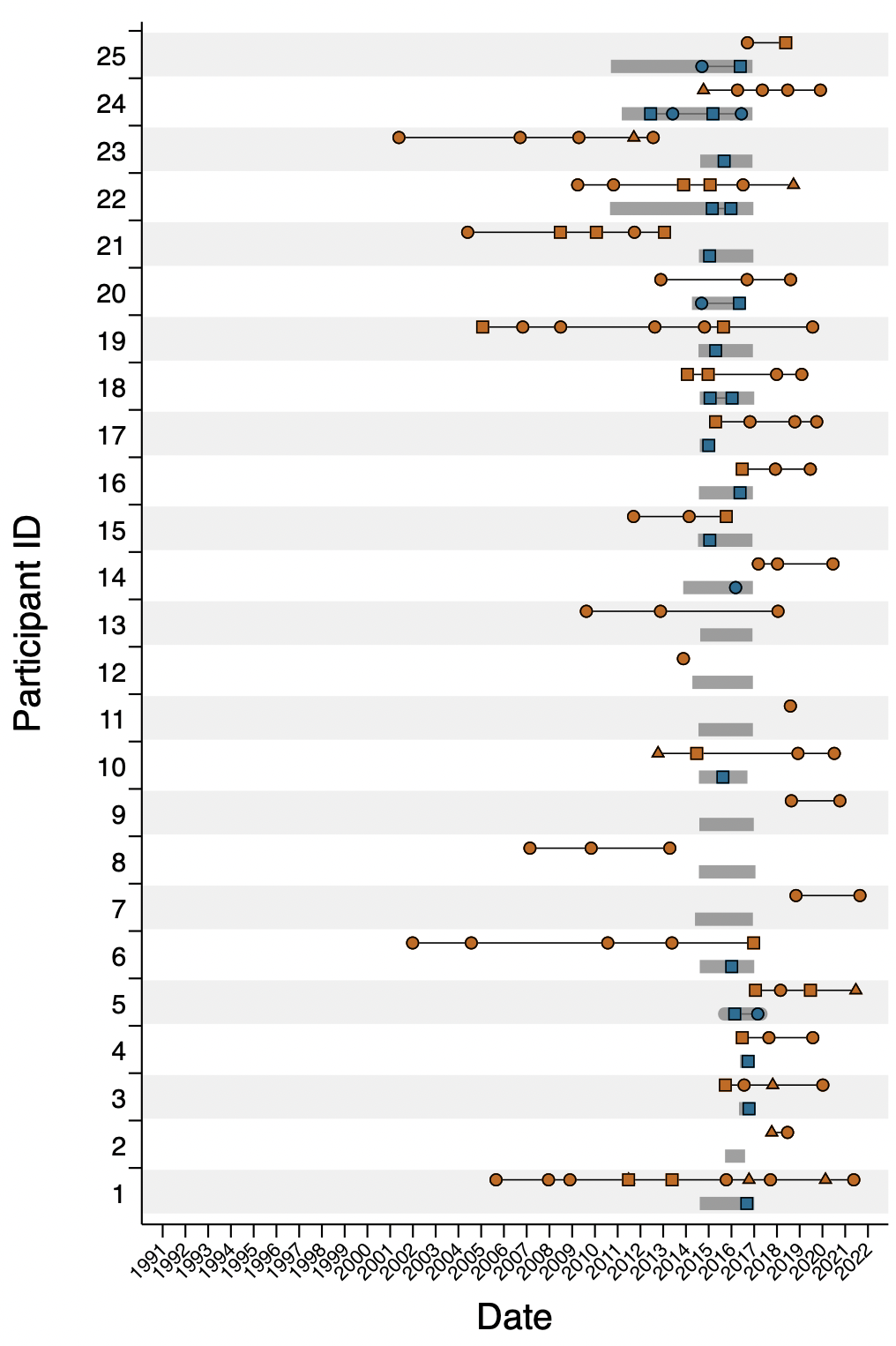
**

**
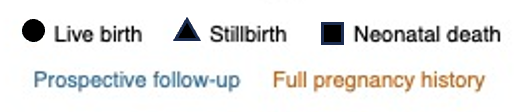
**

**Supplementary Table 3: Participant characteristics by enrollment status in pregnancy history survey**

|  | **Enrolled (n=76)** | **Not enrolled (n=19)** | **Fisher's exact test p-value** |
| --- | --- | --- | --- |
| **Age** |  |  |  |
| <20 | 28 (36.8) | 5 (26.3) |  |
| 20-35 | 45 (59.2) | 13 (68.4) |  |
| >35 | 3 (3.9) | 1 (5.3) | 0.594 |
| **Parity** |  |  |  |
| 1 | 24 (31.6) | 6 (31.6) |  |
| 2 | 14 (18.4) | 3 (15.8) |  |
| 3 | 16 (21.1) | 3 (15.8) |  |
| 4 | 8 (10.5) | 4 (21.1) |  |
| ≥5 | 14 (18.4) | 3 (15.8) | 0.818 |
| **Education** |  |  |  |
| No education | 56 (74.7) | 16 (84.2) |  |
| Some education | 19 (25.3) | 3 (15.8) | 0.547 |
| **Literacy** |  |  |  |
| No | 54 (71.1) | 15 (78.9) |  |
| Yes | 22 (28.9) | 4 (21.1) | 0.577 |
| **Delivery location** |  |  |  |
| Home | 37 (50.7) | 10 (52.6) |  |
| Health facility | 36 (49.3) | 9 (47.4) | >0.999 |

**Supplementary Figure 2: Difference in days between dates of pregnancy outcome for the prospective data vs. pregnancy history survey for outcomes matched within ±30 days (n=124 outcomes; n=62 per source)**


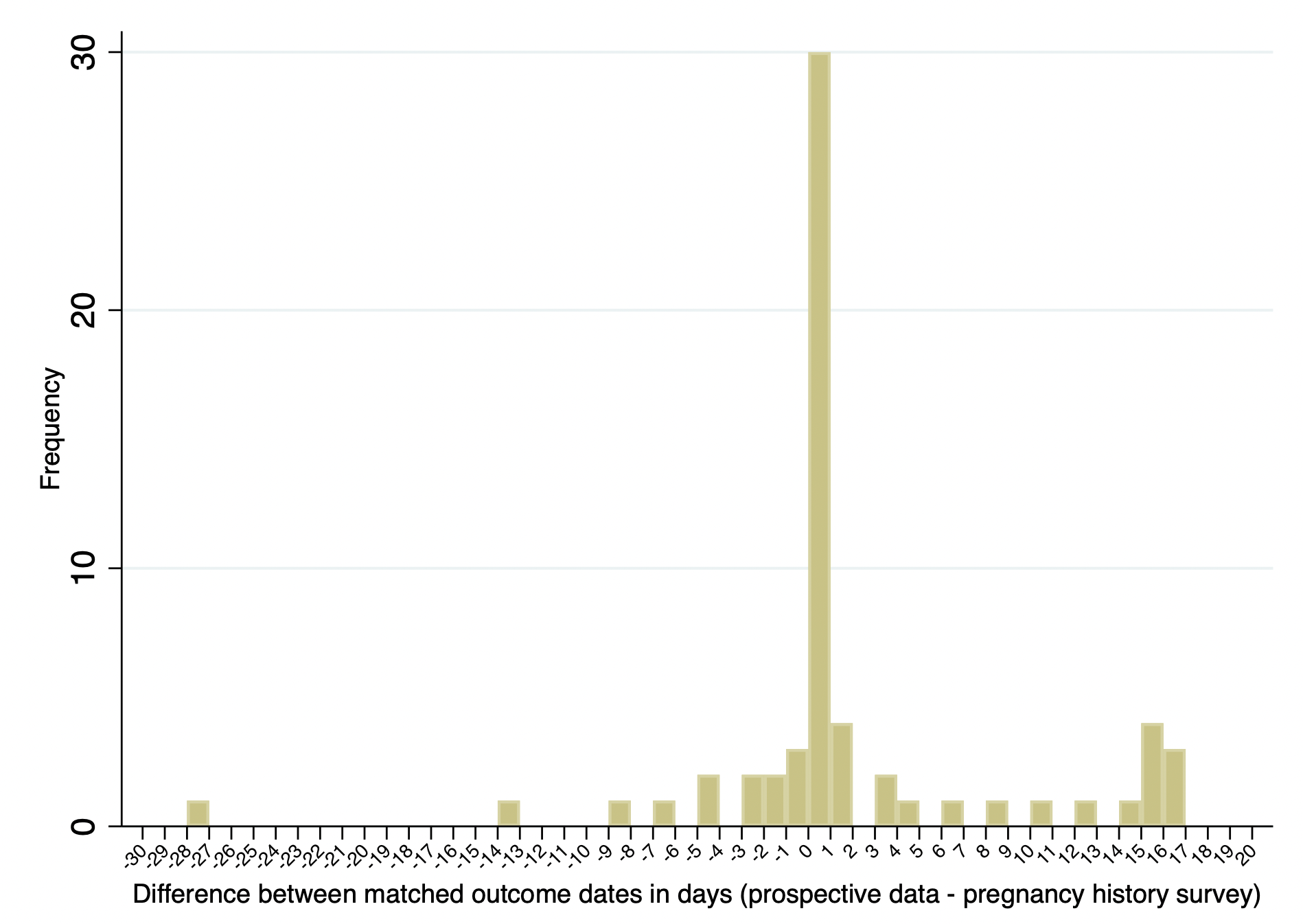


**Supplementary Table 4: Misclassification of stillbirths and neonatal deaths by participant characteristics among 62 pregnancy outcomes matched by date within one month**

|  | **Correct classification (n=57)** | **Misclassification (n=5)** | **Fisher's exact test p-value** |
| --- | --- | --- | --- |
| **Age (years)** |  |  |  |
| <20 | 21 (36.8) | 1 (20.0) |  |
| 20-35 | 36 (63.2) | 3 (60.0) |  |
| >35 | 0 (0.0) | 1 (20.0) | 0.085 |
| **Parity** |  |  |  |
| 0 | 19 (33.3) | 2 (40.0) |  |
| 1 | 11 (19.3) | 1 (20.0) |  |
| ≥2 | 27 (47.4) | 2 (40.0) | >0.999 |
| **Education** |  |  |  |
| No education | 39 (68.4) | 5 (100.0) |  |
| Some education | 18 (31.6) | 0 (0.0) | 0.309 |
| **Antenatal care** |  |  |  |
| <4 visits | 30 (56.6) | 4 (100.0) |  |
| ≥4 visits | 23 (43.4) | 0 (0.0) | 0.140 |
| **Place of delivery** |  |  |  |
| Home | 29 (50.9) | 2 (40.0) |  |
| Health facility | 24 (42.1) | 2 (40.0) |  |
| Don't know | 4 (7.0) | 1 (20.0) | 0.461 |
| **Infant sex** |  |  |  |
| Male | 26 (45.6) | 2 (40.0) |  |
| Female | 31 (54.4) | 3 (60.0) | >0.999 |

**Supplementary Table 5: Pregnancy outcomes in the prospective and pregnancy history survey data after matching outcomes by date within ±30 days, ±60 days, ±100 days, ±365 days, and without restriction***

|  | **Prospective data** | | |
| --- | --- | --- | --- |
| **±30-day match:**  **Validity outcome data (n=62)~** | **Live birth that survived to 28 days** | **Stillbirth** | **Live birth followed by neonatal death** |
| Live birth, survived >28 days | 43 (100.0) | 0 (0.0) | 0 (0.0) |
| Stillbirth | 0 (0.0) | 5 (55.6) | 1 (10.0) |
| Neonatal death | 0 (0.0) | 4 (44.4) | 9 (90.0) |
| **±60-day match**  **Validity outcome data (n=73)** |  |  |  |
| Live birth, survived >28 days | 46 (97.9) | 1 (8.3) | 0 (0.0) |
| Stillbirth | 0 (0.0) | 6 (50.0) | 2 (14.3) |
| Neonatal death | 1 (2.1) | 5 (41.7) | 12 (85.7) |
| **±100-day match**  **Validity outcome data (n=79)** |  |  |  |
| Live birth, survived >28 days | 48 (98.0) | 1 (7.7) | 1 (6.3) |
| Stillbirth | 0 (0.0) | 6 (46.2) | 2 (12.5) |
| Neonatal death | 1 (2.0) | 6 (46.2) | 13 (81.3) |
| **±365-day match**  **Validity outcome data (n=96)** |  |  |  |
| Live birth, survived >28 days | 55 (94.8) | 1 (6.7) | 4 (17.4) |
| Stillbirth | 1 (1.7) | 8 (53.3) | 3 (13.0) |
| Neonatal death | 2 (3.5) | 6 (40.0) | 16 (69.6) |
| **Unrestricted match**  **Validity outcome data (n=104)** |  |  |  |
| Live birth, survived >28 days | 57 (91.9) | 1 (5.9) | 5 (20.0) |
| Stillbirth | 3 (4.8) | 9 (52.9) | 3 (12.0) |
| Neonatal death | 2 (3.2) | 7 (41.2) | 17 (68.0) |
| * For matches >±100 days, some women reported multiple outcomes in the pregnancy history survey that matched with a single outcome from the prospective data; in these cases, we considered the matched outcomes as those with the closest set of dates.  ~ These are the same data from Table 3. They are repeated here to allow for comparison to the other matches. | | | |
